# Hepatic MyD88 regulates liver inflammation by altering synthesis of oxysterols

**DOI:** 10.1152/ajpendo.00082.2019

**Published:** 2019-04-30

**Authors:** Charlotte Lefort, Matthias Van Hul, Nathalie M. Delzenne, Amandine Everard, Patrice D. Cani

**Affiliations:** Metabolism and Nutrition Research Group, Louvain Drug Research Institute, UCLouvain, Université Catholique de Louvain and Walloon Excellence in Life Sciences and Biotechnology, Brussels, Belgium

**Keywords:** bile acids, 25-hydroxycholesterol, immunity, inflammation, MyD88, oxysterols

## Abstract

This study aimed to investigate the function of hepatic myeloid differentiation primary response gene 88 (MyD88), a central adaptor of innate immunity, in metabolism. Although its role in inflammation is well known, we have recently discovered that MyD88 can also mediate energy, lipid, and glucose metabolism. More precisely, we have reported that mice harboring hepatocyte-specific deletion of *MyD88* (Myd88^ΔHep^) were predisposed to glucose intolerance, liver fat accumulation, and inflammation. However, the molecular events explaining the onset of hepatic disorders and inflammation remain to be elucidated. To investigate the molecular mechanism, Myd88^ΔHep^ and wild-type (WT) mice were challenged by two complementary approaches affecting liver lipid metabolism and immunity. The first approach consisted of a short-term exposure to high-fat diet (HFD), whereas the second was an acute LPS injection. We discovered that upon 3 days of HFD Myd88^ΔHep^ mice displayed an increase in liver weight and liver lipids compared with WT mice. Moreover, we found that bile acid and oxysterol metabolism were deeply affected by the absence of hepatic MyD88. Our data suggest that the negative feedback loop suppressing bile acid synthesis was impaired (i.e., ERK activity was decreased) in Myd88^ΔHep^ mice. Finally, the predisposition to inflammation sensitivity displayed by Myd88^ΔHep^ mice may be caused by the accumulation of 25-hydroxycholesterol, an oxysterol linked to inflammatory response and metabolic disorders. This study highlights the importance of MyD88 on both liver fat accumulation and cholesterol-derived bioactive lipid synthesis. These are two key features associated with metabolic syndrome. Therefore, investigating the regulation of hepatic MyD88 could lead to discovery of new therapeutic targets.

## INTRODUCTION

The liver is an essential organ that is involved in many metabolic features such as lipid and glucose metabolism, xenobiotic detoxification, and bile production. Over the last decade the incidence of liver diseases has increased, becoming epidemic. The most common is nonalcoholic fatty liver disease, which is clearly linked with the increased prevalence of obesity and its comorbidities (17). Different drivers are contributing to liver diseases, including diet, innate immunity stimulation, gut microbiota, and host genetic background (17, 19, 23, 26). Although the numbers of metabolic-related liver disorders are rising, the molecular mechanisms and triggering factors are not yet fully deciphered.

Myeloid differentiation primary response gene 88 (MyD88) is a key player in the innate immune system. It is the main adaptor protein not only of IL-1 and IL-18 receptors but also of all Toll-like receptors (TLRs) except TLR3. TLR stimulation initiates an immune response and leads to proinflammatory cytokine production by activating several proteins and transcription factors such as c-Jun NH_2_-terminal kinase (JNK) and nuclear factor-κB (NF-κB) (21). MyD88 is thus considered a central hub of the inflammatory signaling cascades. Although its role in inflammation and immunity is well established, we have recently shown that MyD88 can also modulate metabolism (e.g., energy, glucose, and lipid metabolism) (8, 9). Indeed, we reported that intestinal epithelial MyD88 acts as a nutrient sensor and controls energy expenditure (9). More recently we have discovered that mice harboring hepatocyte-specific deletion of *MyD88* (Myd88^∆Hep^) are predisposed to liver fat accumulation and inflammation (8). Besides this observation, Myd88^∆Hep^ mice also exhibited altered gut microbiota and bile acid metabolism (8). However, this phenotype has only been studied upon a prolonged exposure to a high-fat diet (HFD), and the molecular events explaining the onset of hepatic disorders and inflammation remain to be elucidated.

Therefore, this study aimed to investigate the mechanisms behind the Myd88^∆Hep^ phenotype in order to find new putative targets responsible for the onset of metabolic liver disorders. Hence, we designed two complementary approaches known to challenge liver lipid metabolism and immunity. The first consists of a short-term exposure to HFD and the second of an acute injection of lipopolysaccharide (LPS), the major component of the outer membrane of gram-negative bacteria.

## MATERIALS AND METHODS

### Mice

#### Generation of Myd88^∆Hep^ mice.

Hepatocyte *Myd88*-deleted (Myd88^∆Hep^) mice were generated as previously described in Duparc et al. (8). Briefly, mice harboring *Cre* recombinase expressed under the *albumin* promoter (*Albumin*-Cre) (C57BL/6 background; Jackson Laboratory, Bar Harbor, ME) were crossed with mice bearing a *loxP*-flanked *Myd88* allele (C57BL/6 background; Jackson Laboratory). Genotyping and validation of the deletion in the offspring were performed as described in Duparc et al. (8). The control mice were wild-type (WT) littermates harboring the *Myd88 loxP*-flanked allele but not the *Cre* recombinase.

Mice were housed in a controlled environment (12-h daylight cycle, lights off at 6 PM) and in specific pathogen-free conditions in groups of two mice per cage (filter-top cages), with free access to irradiated food and autoclaved water. The mice were fed a normal control diet (AIN93Mi; Research Diets, New Brunswick, NJ).

#### Short-term high-fat diet experiment.

A cohort of 10-wk-old male Myd88^∆Hep^ and WT mice were fed either a control diet (CT) (10% fat, AIN93Mi; Research Diets) (WT-CT or Myd88^∆Hep^-CT) or a HFD (60% fat, D12492i; Research Diets) (WT-HFD or Myd88^∆Hep^-HFD) for 3 days.

#### LPS injection experiment.

A cohort of CT-fed male Myd88^∆Hep^ and WT mice were injected intraperitoneally with either 300 µg/kg LPS solution (LPS from *Escherichia coli* O55:B5; Sigma L2880) or saline solution (CT). Mice were euthanized 4 h after the injection.

### Tissue Sampling

At the end of the treatment period, fed animals were anesthetized with isoflurane (Forene; Abbott) and blood was sampled from the portal vein. After blood sampling mice were killed by cervical dislocation, and both liver and cecum were immediately immersed in liquid nitrogen and stored at −80°C for further analysis.

### RNA Preparation and Real-Time qPCR Analysis

Total RNA was prepared from tissues with TriPure Reagent (Roche). Quantification and integrity analysis of total RNA were performed by running 1 μl of each sample on an Agilent 2100 Bioanalyzer (Agilent RNA 6000 Nano Kit; Agilent). The cDNA was prepared by reverse transcription, and real-time qPCR was performed as previously described by Everard et al. (9). *Rpl19* RNA was chosen as housekeeping gene. Sequences of the primers used for real-time qPCR are shown in Table 1.

**Table 1.** Primers used for real-time qPCR

Gene	Forward Primer Sequence (5′–3′)	Reverse Primer Sequence (5′–3′)
*Rpl19*	GAAGGTCAAAGGGAATGTGTTCA	CCTTGTCTGCCTTCAGCTTGT
*Il6*	ACAAGTCGGAGGCTTAATTACACAT	TTGCCATTGCACAACTCTTTTC
*Cd11c*	ACGTCAGTACAAGGAGATGTTGGA	ATCCTATTGCAGAATGCTTCTTTACC
*F4/80*	TGACAACCAGACGGCTTGTG	GCAGGCGAGGAAAAGATAGTGT
*Il1b*	TCGCTCAGGGTCACAAGAAA	CATCAGAGGCAAGGAGGAAAAC
*Tnfa*	TCGAGTGACAAGCCTGTAGCC	TTGAGATCCATGCCGTTGG
*Hmgcr*	CCTGACACTGAACTGAAGCG	TCTTTCCAGAACACAGCACG
*Cyp7a1*	GGGATTGCTGTGGTAGTGAGC	GGTATGGAATCAACCCGTTGTC
*Cyp27a1*	TCTGGCTACCTGCACTTCCT	GTGTGTTGGATGTCGTGTCC
*Cyp8b1*	GATCCGTCGCGGAGATAAGG	CGGGTTGAGGAACCGATCAT
*Cyp7b1*	TAGGCATGACGATCCTGAAA	TCTCTGGTGAAGTGGACTGAAA

### Western Blotting

For detection of proteins, liver tissues were homogenized with TissueLyser II (Qiagen) in ice-cold buffer [in mM: 20 Tris, 270 sucrose, 5 EGTA, 1 EDTA, 1 sodium orthovanadate, 50 sodium β-glycerophosphate, 5 sodium pyrophosphate, 50 sodium fluoride, and 1 1,4-dithiothreitol, with 1% Triton X-100 and 10% protease inhibitor cocktail 10× (Roche Applied Science, Vilvoorde, Belgium)]. Homogenates were centrifuged at 10,000 *g* for 10 min at 4°C. Supernatants were immediately stored at −20°C. Equal amounts of proteins were separated by SDS-PAGE and transferred to nitrocellulose membranes. Membranes were incubated overnight at 4°C with antibodies diluted in Tris-buffered saline-Tween 20 containing 1% bovine serum albumin: JNK (1:1,000; 9252S, Cell Signaling), phosphorylated (p-)JNK (1:200; 9251S, Cell Signaling), ERK (1:1,000; 4695S, Cell Signaling), and p-ERK (1:1,000; 9101S, Cell Signaling). The loading control was β-actin (1:10,000; ab6276, Abcam). The difference in protein loading is taken into account when signal quantification is analyzed. Signal quantification was acquired with an Amersham Imager 600 (GE Healthcare) and analyzed by ImageQuant TL software.

### Liver Lipid Content

Total lipid content was measured in the liver tissue after extraction with chloroform-methanol according to the Folch method, as previously described by Duparc et al. (8) and Folch et al. (10). Triglyceride and cholesterol concentrations were measured with kits coupling enzymatic reaction and spectrophotometric detection of reaction end products (Diasys Diagnostic and Systems, Holzheim, Germany).

### Lipidomics Analysis

Lipid measurements were performed in collaboration with Biocrates (Innsbruck, Austria).

Bile acid quantification was performed with a commercial Bile Acids Kit from Biocrates. A selective reverse-phase LC-MS/MS analysis method in negative ion multiple reaction monitoring (MRM) detection mode was used to measure bile acid levels. Analyses were performed via LC-electrospray ionization (ESI)-MS/MS with a SCIEX 4000 QTRAP (SCIEX, Darmstadt, Germany) instrument. Seven-point external calibration curves and 10 stable isotope-labeled internal standards were used.

Prostaglandins were purified from 20 µl of biological sample with a methanolic protein precipitating solution. A MRM mode using negative ESI was applied to detect prostaglandins. An online solid phase extraction-HPLC-MS/MS on a SCIEX 5500 QTRAP (SCIEX) instrument was used to carry out the analysis. Several deuterated metabolites were used as internal standards; quantitation was performed with a seven-point calibration.

Extractions of sterols and oxysterols from samples were performed with methanol and the Biocrates Kit filter plate. A UHPLC-MS/MS with MRM in positive mode using a SCIEX API 5500 QTRAP (SCIEX) instrument with ESI was applied to determine the concentration of sterols and oxysterols.

All data were quantified with MS software (ThermoFisher Scientific Xcalibur) and Biocrates MetIDQ software.

### Ethics Statement

All mouse experiments were reviewed and approved by and performed in accordance with the guidelines of the local ethics committee for animal care of the Health Sector of the Université Catholique de Louvain under the specific agreement numbers 2014/UCL/MD/022 and 2017/UCL/MD/005. Housing conditions were as specified by the Belgian Law of 29 May 2013 regarding the protection of laboratory animals (agreement number LA1230314). Every effort was made to minimize animal pain, suffering, and distress.

### Statistical Analysis

Data are expressed as means ± SE. Differences between more than two groups were assessed by two-way ANOVA, followed by the Tukey post hoc test. Data were analyzed with GraphPad Prism (GraphPad Software).

## RESULTS

### Hepatocyte Myd88-Deleted Mice Are Predisposed to Accumulate Lipids in the Liver

**Fig. 1.**
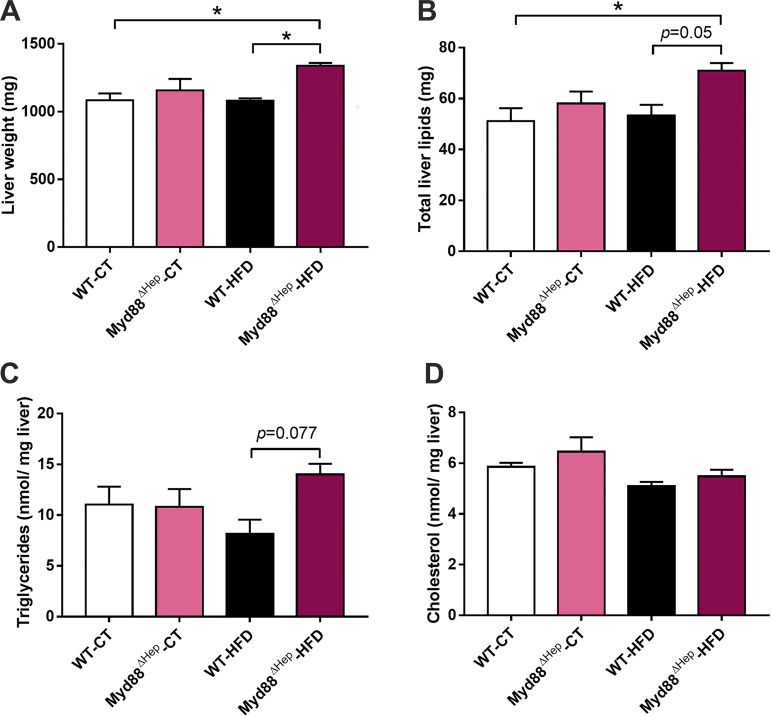
Hepatocyte myeloid differentiation primary response gene 88 (*Myd88*) deletion (Myd88^ΔHep^) worsens high-fat diet (HFD)-induced fat accumulation. *A*: mean liver weight. *B*: total lipid levels measured in the liver. *C*: liver triglyceride measurement. *D*: liver cholesterol measurement. Data are presented as means ± SE (*n* = 6–9 mice). CT, control diet; WT, wild type. Significantly different according to 2-way ANOVA followed by Tukey post hoc test: *P < 0.05.

In the present study, we exposed WT and Myd88^∆Hep^ mice to 3 days of HFD. We hypothesized that ingestion of a hyperlipidic diet for a short-term period would trigger bioactive lipid production without the potential compensation effect observed during a prolonged HFD exposure (i.e., 8 wk). Interestingly, upon 72 h of HFD exposure Myd88^∆Hep^ mice showed a significant increase in liver weight compared with WT mice (Fig. 1*A*). Accordingly, both liver lipid and triglyceride levels were markedly increased in Myd88^∆Hep^ mice compared with WT mice under HFD (*P* < 0.01, *t*-test; Fig. 1, *B* and *C*). However, we did not observe any modification of liver cholesterol levels (Fig. 1*D*).

Altogether, these data indicate that hepatocyte MyD88 modulates liver lipid storage after a short exposure to HFD.

### Myd88 Deletion Strongly Modifies Bile Acid Metabolism

Our first data confirmed an impairment of liver lipid regulation in Myd88^∆Hep^ mice also after a short HFD exposure. Since we previously reported that bile acid metabolism was altered in Myd88^∆Hep^ mice during long-term HFD (8 wk) (8), we wondered whether bile synthesis was already affected under short-term HFD in Myd88^∆Hep^ mice.

**Fig. 2.**
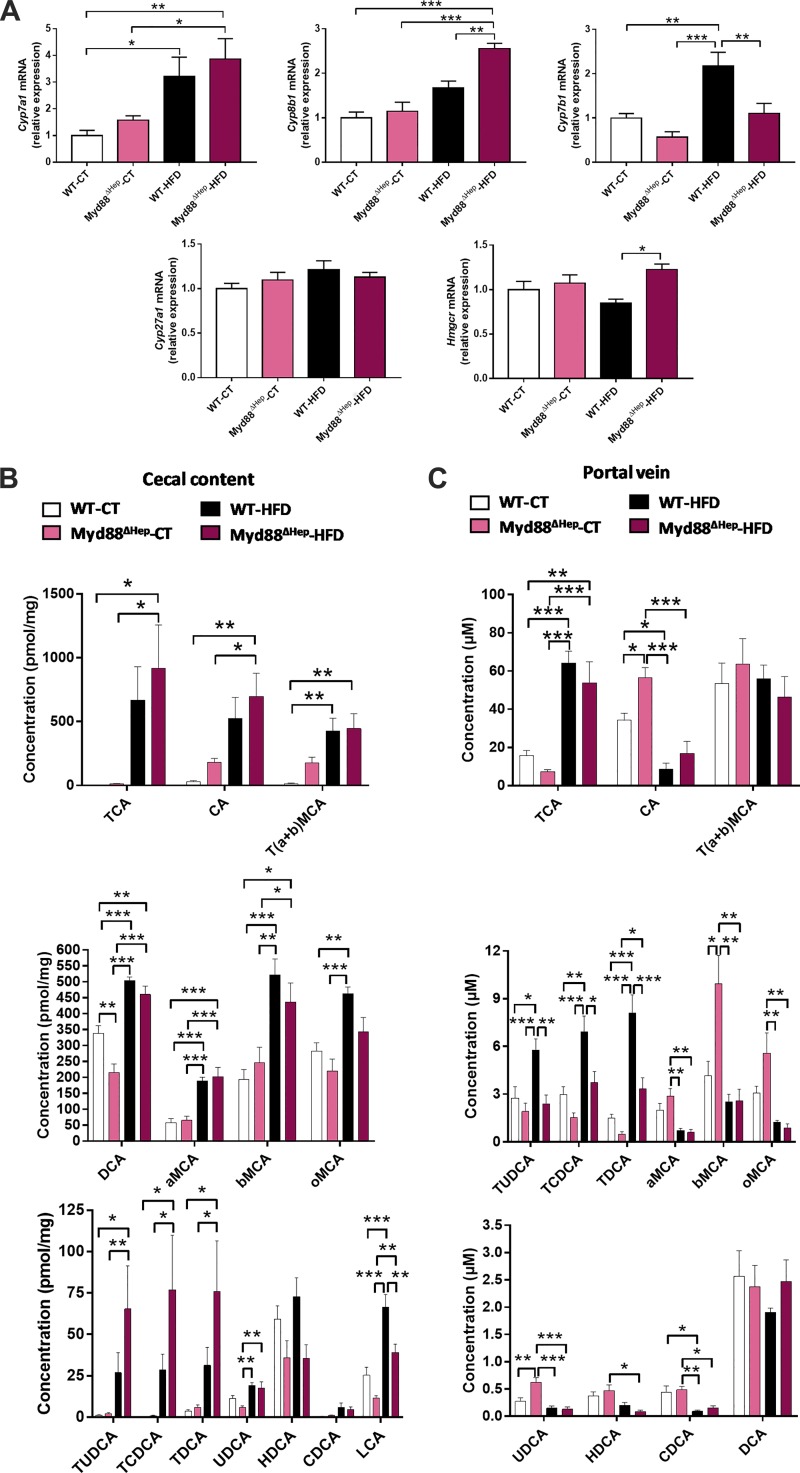
Hepatocyte myeloid differentiation primary response gene 88 (*Myd88*) deletion (Myd88^ΔHep^) profoundly impacts bile acid metabolism. *A*: liver mRNA expression of bile acid synthesis enzymes measured by real-time qPCR in wild-type (WT) and Myd88^ΔHep^ mice fed with either control diet (CT) or high-fat diet (HFD). *B*: cecal bile acid content. *C*: plasma (portal vein) bile acid content. Data are presented as means ± SE (*n* = 6–9 mice). CA, cholic acid; CDCA, chenodeoxycholic acid; DCA, deoxycholic acid; HDCA, hyodeoxycholic acid; LCA, lithocholic acid; MCA, muricholic acid; T, taurine; UDCA, ursodeoxycholic acid; a, α, b, β, o, ω conjugated species. Significantly different according to 2-way ANOVA followed by Tukey post hoc test: *P < 0.05, **P < 0.01, ***P < 0.001.

First, we analyzed the expression of key enzymes regulating bile acid production, and we observed that the enzyme synthesizing cholesterol, *Hmgcr,* was significantly increased in Myd88^∆Hep^-HFD compared with WT-HFD mice (Fig. 2*A*). *Cyp8b1* mRNA expression followed the same expression pattern (Fig. 2*A*). Additionally, the rate-limiting enzyme of bile acid synthesis, *Cyp7a1,* was also overexpressed in Myd88^∆Hep^-CT versus WT-CT mice (*P* < 0.05, *t*-test; Fig. 2*A*). It is worth noting that among all the enzymes measured, *Cyp7b1* was the only one to be found significantly decreased in Myd88^∆Hep^ mice in both conditions (Fig. 2*A*).

Second, to further explore the impact of MyD88 on bile acid regulation, we examined the bile acid profile in the cecal content and the portal vein blood of Myd88^∆Hep^ and WT mice (Fig. 2, *B* and *C*). The results showed that, in general, all mice exposed to HFD had an overall increase in bile acid concentration in both portal vein and cecal content. Of note, the primary bile acids [cholic acid (CA), chenodeoxycholic acid (CDCA), a- and b-muricholic acid (MCA)] and some secondary bile acids [ursodeoxycholic acid (UDCA), hyodeoxycholic acid (HDCA), and o-MCA] from the portal vein were the only bioactive lipids measured displaying a lower level upon HFD exposure (Fig. 2*C*). Strikingly, although HFD has a clear impact on bile acid metabolism, the alteration pattern of bile acid levels in Myd88^∆Hep^ mice was almost identical independently of diet and was clearly different compared with WT mice. Hence our data indicate that the genotype has a stronger influence on bile acid pathways than diet per se. Indeed, in the portal vein the detection of conjugated bile acids [e.g., TDCA, TCDCA, TUDCA (in HFD), TCA (in CT, *P* < 0.01, Mann-Whitney test)] were mostly decreased (Fig. 2*C*). In line with this result, a higher proportion of conjugated bile acids were measured in the cecal content of Myd88^∆Hep^ mice (Fig. 2*B*).

It is also worth noting that in cecal content the levels of secondary bile acids were mainly lower in Myd88^∆Hep^ mice (Fig. 2*B*). Indeed, under CT diet UDCA (*P* < 0.05, *t-*test) and DCA were markedly decreased in Myd88^∆Hep^ mice, whereas under HFD HDCA and o-MCA (*P* < 0.05, *t-*test) and lithocholic acid concentrations were significantly lower. Finally, CA was the only bile acid following the same trend in both cecal content and portal vein (Fig. 2,*B* and *C*). Interestingly, a strong elevation of CA concentration was observed in Myd88^∆Hep^-CT compared with WT-CT mice. Upon HFD, the levels were also elevated in Myd88^∆Hep^ mice, but this effect did not reach significance.

Altogether, this set of data confirms a clear impact of MyD88 on the control of bile acid production and levels in both cecum and blood.

### Enterohepatic Loop Repressing Cyp7a1 and Cyp8b1 Is Impaired in Myd88^∆Hep^ Mice

**Fig. 3.**
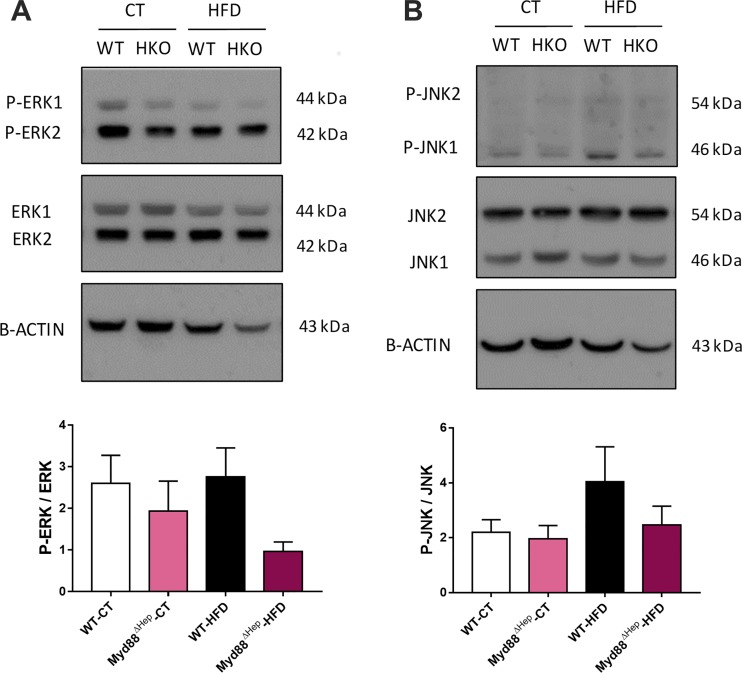
Hepatocyte myeloid differentiation primary response gene 88 (*Myd88*) deletion (Myd88^ΔHep^) affects ERK activity: Western blot analysis of liver protein extracts from Myd88^ΔHep^ and wild-type (WT) mice fed with either control diet (CT) or high-fat diet (HFD). Ratio quantification is measured by densitometry and is expressed relative to β-actin. *A*: expression of ERK1/2 and its active form, phosphorylated (p-)ERK1/2. *B*: expression of JNK1/2 and its active form, p-JNK1/2. Quantification data are presented as means ± SE (*n* = 6–7).

Since our data indicated that Myd88^∆Hep^ mice overexpressed *Cyp7a1* and *Cyp8b1* mRNA, we wondered whether the enterohepatic feedback loop controlling bile acid synthesis could be altered in Myd88^∆Hep^ compared with WT mice. The regulation of bile acid production is mediated by the stimulation of intestinal fibroblast growth factor 15 (FGF15; FGF19 in humans) expression thanks to farnesoid X receptor activation by specific bile acids (14). FGF15 then reaches the liver through the portal vein and activates the hepatic fibroblast growth factor receptor 4 (FGFR4) (14). FGFR4 stimulation induces the phosphorylation of JNK and ERK to activate them. In turn, p-JNK and p-ERK promote the repression of CYP7A1 and CYP8B1, suppressing bile acid production. It is important to note that the proper molecular mechanism underlying this hepatic signaling pathway still remains elusive (16). Because we have previously demonstrated that Myd88^∆Hep^ mice express normal levels of both FGF15 and FGFR4 (8), we hypothesized that the deletion of *Myd88* would impact this feedback loop downstream of FGF15/FGFR4, at the hepatic level. We discovered that p-ERK was lower in Myd88^∆Hep^-HFD versus WT-HFD mice (*P* < 0.05, *t-*test), whereas a trend was observed for p-JNK (Fig. 3, *A* and *B*, respectively).

Taken together, a decreased activity of ERK in Myd88^∆Hep^-HFD mice highlights the importance of MyD88 in modulating the hepatic feedback loop responsible for the repression of bile acid synthesis. These results are in line with the increase in mRNA expression of bile acid synthesis enzymes.

### Predisposition to Liver Inflammation Observed in Myd88^∆Hep^ Mice Is Linked with Altered Oxysterol Metabolism

Although one may intuitively expect a decreased inflammatory response in mice lacking hepatic MyD88, we have recently reported that Myd88^∆Hep^ mice are in fact more sensitive to inflammation (8).

**Fig. 4.**
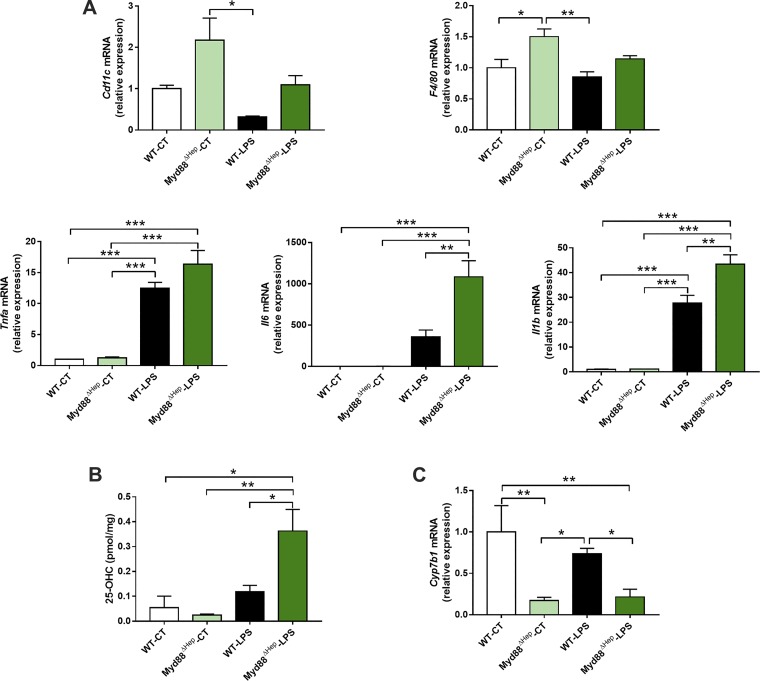
Myeloid differentiation primary response gene 88 (*Myd88*)-deleted mice are predisposed to inflammation, presumably due to the alteration of oxysterol metabolism. *A*: liver mRNA expression of inflammatory markers measured by real-time qPCR in wild-type (WT) and Myd88^ΔHep^ mice injected with either saline solution [control (CT)] or LPS. *B*: liver 25-hydroxycholesterol (25-OHC) concentration. *C*: liver *Cyp7b1* mRNA expression measured by real-time qPCR. Data are presented as means ± SE (*n* = 3–5 mice). Significantly different according to 2-way ANOVA followed by Tukey post hoc test: *P < 0.05, **P < 0.01, ***P < 0.001.

**Fig. 5.**
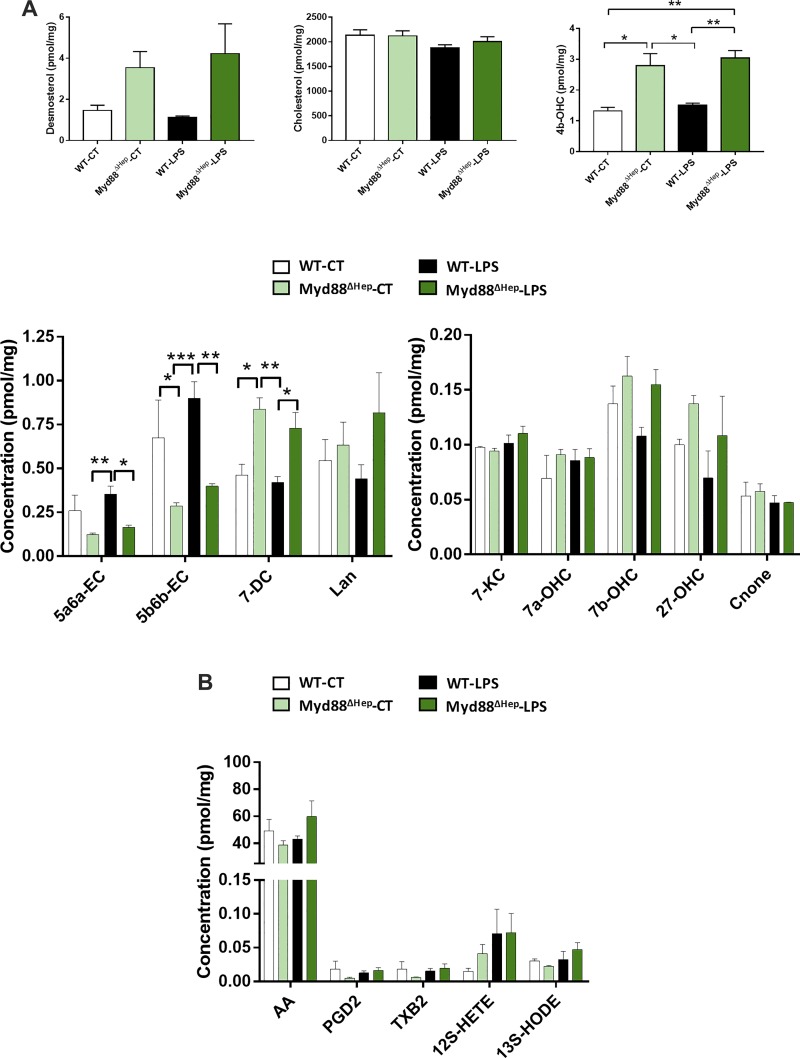
Hepatocyte myeloid differentiation primary response gene 88 (*Myd88*) deletion (Myd88^ΔHep^) strongly impacts cholesterol-derived bioactive compound synthesis in the liver. *A*: liver measurements of cholesterol and its relative compounds in wild-type (WT) and Myd88^ΔHep^ mice injected with either saline solution [control (CT)] or LPS. *B*: prostaglandins and relative molecule measurements in liver extract of Myd88^ΔHep^ and WT mice injected with either saline solution or LPS. Data are presented as means ± SE (*n* = 3–5 mice). 4b-OHC, 4β-hydroxycholesterol; 5a6a-EC, 5α,6α-epoxycholesterol; 5b6b-EC, 5β,6β-epoxycholesterol; 7-DC, 7-dehydrocholesterol; 7-KC, 7-ketocholesterol; 7a-OHC, 7α-hydroxycholesterol; 7b-OHC; 7β-hydroxycholesterol, 27-OHC, 27-hydroxycholesterol; Cnone, 7α-hydroxycholestenone; Lan, lanosterol. AA, arachidonic acid; PGD2, prostaglandin D_2_; TXB2, thromboxane B2; 12S-HETE, 12(*S*)-hydroxy-5*Z*,8*Z*,10*E*,14*Z*-eicosatetraenoic acid; 13S-HODE, 13(*S*)-hydroxy-9*Z*,11*E*-octadecadienoic acid. Significantly different according to 2-way ANOVA followed by Tukey post hoc test: *P < 0.05, **P < 0.01, ***P < 0.001.

To investigate the molecular action behind this inflammation sensitivity, Myd88^∆Hep^ and WT mice were injected with LPS. When challenged with LPS, Myd88^∆Hep^ mice exhibited a worsened inflammation compared with WT. Indeed, the hepatic mRNA expressions of *Tnfa*, *Il6*, and *Il1b* were markedly increased (fold change of 1.3, 3, and 1.5, respectively) in Myd88^∆Hep^-LPS compared with WT-LPS mice (Fig. 4*A*). In addition, *Cd11c* and *F4/80* mRNA expression levels tended to be more elevated in Myd88^∆Hep^ compared with WT mice (Fig. 4*A*). Since liver lipid regulation was affected in mice deleted for *Myd88* in the hepatocyte, we measured two important families of bioactive lipids contributing to host homeostasis and to the regulation of inflammation. The first family includes prostaglandins, whereas the second is related to cholesterol derivatives, namely oxysterols. Although it is well established that prostaglandins mediate inflammation, we did not find relevant alterations between Myd88^∆Hep^ and WT mice (Fig. 5*B*). In contrast, the levels of several oxysterols were deeply altered by the absence of hepatic MyD88 (Fig. 5*A*). Interestingly, among all the oxysterols measured we found that 25-hydroxycholesterol (25-OHC) was the only one following the same pattern of expression as proinflammatory cytokines (Fig. 4*B*). It is important to mention that 25-OHC has been described as a modulator of immune functions and inflammatory markers (5). To further investigate this finding, we measured the main enzyme degrading 25-OHC (i.e., *Cyp7b1*) and found that its expression was significantly decreased in Myd88^∆Hep^ mice in both conditions (Fig. 4*C*).

Therefore, these results suggest that hepatocyte deletion of *Myd88* predisposes mice to inflammation and this effect is strongly linked with an alteration of oxysterol metabolism.

## DISCUSSION

The incidence of metabolic disorders is constantly rising and is often associated with liver fat accumulation (17). In the present study, we discovered that mice lacking MyD88 in the hepatocytes are prone to liver fat accumulation. Indeed, when challenged with a HFD for 3 days, Myd88^∆Hep^ mice displayed higher liver weight and an elevation of lipid and triglyceride contents. Next, we demonstrated that MyD88 controls the synthesis of cholesterol-derived bioactive lipids in both HFD and LPS challenge conditions. Moreover, we discovered that the profiles of bile acids or their precursors, namely oxysterols, were profoundly affected by the absence of hepatic MyD88.

Unexpectedly, liver levels of cholesterol were not higher in any experiments, even though the enzyme responsible for its synthesis was increased (HFD study) and its precursor, desmosterol, tended to be more elevated in Myd88^∆Hep^ mice (Fig. 5*A*; LPS study). Given that Myd88^∆Hep^ mice displayed an increased rate of bile acid synthesis, our results suggest that the putative excess of cholesterol might be directly metabolized into oxysterols and/or bile acids. Indeed, intracellular accumulation of cholesterol is toxic, and one way to remove extra cholesterol from the liver is to increase bile acid and oxysterol synthesis (24).

Our data show that the feedback loop controlling bile acid production was altered. Indeed, the main enzymes repressed by the negative feedback loop involved in bile acid synthesis were overexpressed in Myd88^∆Hep^ mice, suggesting that the FGF15-FGFR4-JNK-ERK-CYP7A1/CYP8B1 pathway was affected by *Myd88* deletion. Because we have previously demonstrated that FGF15 and FGFR4 levels were normal in Myd88^∆Hep^ compared with WT mice (8), we focused our attention on the downstream effectors regulating this signaling cascade. We found that activated ERK, p-ERK, which has been described to inhibit CYP7A1 and CYP8B1 in parallel with p-JNK (16, 18), was lower in Myd88^∆Hep^ mice fed with HFD compared with WT mice. Since *Cyp8b1* is strongly overexpressed in Myd88^∆Hep^-HFD mice, it might indicate that p-ERK is more potent at inhibiting it than *Cyp7a1*. Finally, the reason why we did not observe significant changes regarding p-JNK could result from the fact that the FGF15-FGFR4 pathway activates ERK strongly than JNK (16).

Collectively, our data support the hypothesis that hepatic MyD88 is involved in the fine-tuning of bile acid synthesis by interacting with the enterohepatic feedback loop.

In line with the fact that hepatic MyD88 mediates cholesterol-derivative bioactive lipid metabolism, we propose that the increased sensitivity to inflammation observed in Myd88^∆Hep^ mice injected with LPS may be due to the accumulation of one specific oxysterol, namely, 25-OHC. In fact, in the last decade this oxysterol has emerged as a regulator of immune cell function by displaying antiviral activities, regulating IgA formation, inducing macrophage foam cell production, and controlling inflammation (see Ref. 5 for review). However, its impact on inflammation is still debated because 25-OHC has been shown to exhibit both pro- and anti-inflammatory properties. On one hand, the production of type 1 interferon by viral or bacterial infection induces 25-OHC production, whereby this oxysterol exerts an anti-inflammatory activity by inhibiting sterol regulatory element-binding protein, resulting in the feedback inhibition of IL-1 family production (12, 22). On the other hand, other studies have reported the importance of 25-OHC to amplify inflammation by promoting some inflammatory gene expression, likely via NF-kB signaling pathways (5, 11, 15). It is also suggested that the mediation of this immune process is complex, and we may speculate that the impact of 25-OHC depends on its concentration, the cell types involved, and the type of receptors promoting its formation (1, 5, 6). Previous findings clearly demonstrated that 25-OHC is induced by LPS (1, 7). Here we found that the accumulation of 25-OHC observed in the liver of Myd88^∆Hep^ mice was even worsened after LPS injection. This is likely due to the decreased expression of its degrading enzyme, *Cyp7b1*, compared with WT mice. Since we observed a significant increase in mRNA expression of *Il1b* and other proinflammatory markers in the liver of Myd88^∆Hep^-LPS mice, we might suggest that at this concentration the anti-inflammatory effect of 25-OHC is probably not effective. However, this assumption warrants further investigations.

Strikingly, a similar decrease in *Cyp7b1* mRNA expression as well as an increase in 25-OHC level have also been reported in the liver of both *ob/ob* and *db/db* mice, strengthening the fact that the dysregulation of hepatic MyD88 might be involved in metabolic syndrome (13).

In addition to the data described above, we have also found an increase in 4β-hydroxycholesterol (4b-OHC) in both basal and challenged conditions in Myd88^∆Hep^ mice (Fig. 5). An elevation of this oxysterol has been associated with indole alleviating liver inflammation after LPS injection (2). Moreover 4b-OHC is a liver X receptor (LXR) agonist, and activation of LXR induces anti-inflammatory effects (20, 25). Besides the increase in 4b-OHC, we found that other putative regulators of LXR activity (i.e., desmosterol, 25-OHC, 5α,6α-epoxycholesterol) were changed in Myd88^∆Hep^ mice (Fig. 5*A*). One key LXR target gene, *Cyp7a1*, was also upregulated. Therefore, although we did not assess precisely the LXR activity, we may hypothesize that MyD88 is involved in the regulation of liver metabolism by affecting various key metabolites such as bile acids and oxysterols, both involved in inflammatory response and glucose and lipid metabolism.

**Fig. 6.**
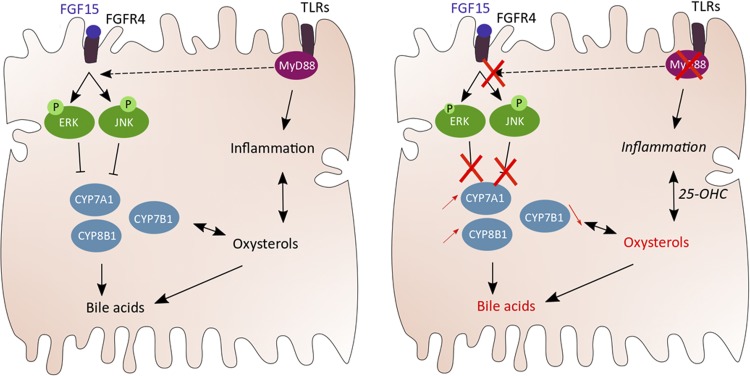
Illustration of the findings and working hypothesis. Our data show that hepatic myeloid differentiation primary response gene 88 (MyD88), in addition to being involved in immunity, can regulate metabolism. In addition, we demonstrate that hepatic MyD88 controls cholesterol-derived bioactive lipid synthesis, presumably by regulating the negative feedback loop inhibiting bile acid production. Finally, an accumulation of 25-hydroxycholesterol (25-OHC) would predispose hepatocyte *Myd88*-deletion (Myd88^∆Hep^) mice to inflammation. Thus we discovered a novel mechanism of interaction between the innate immune system and the synthesis of different bioactive lipids thereby controlling host metabolism. FGF15; fibroblast growth factor 15; FGFR4, fibroblast growth factor receptor 4; JNK, c-Jun NH_2_-terminal kinase; P, phosphorylated; TLRs, Toll-like receptors.

Altogether, our results suggest that hepatocyte-specific *Myd88* deletion predisposed mice to inflammation by altering liver oxysterol levels (Fig. 6).

Finally, emerging evidence has also described an interesting interaction between the liver and the gut microbiota through the gut-liver axis, contributing to liver homeostasis (3, 26). As a matter of fact, modification of bile acids can affect gut microbiota population and proliferation. In turn, these intestinal bacteria are able to mediate bile acid composition by converting primary bile acids into secondary bile acids. These observations highlight a reciprocal regulation between those two key factors (3). In the present study, we found that Myd88^∆Hep^ mice exhibited an enhanced bile acid synthesis. It has been established that an increased bile acid concentration exerts bactericidal effects (4). In accordance with this observation, we have previously shown that Myd88^∆Hep^ mice displayed an altered gut microbiota composition (8). We can thus speculate that the microbial community present in Myd88^∆Hep^ is disturbed. In line with this hypothesis, we found that the concentration of secondary bile acids in the cecum was lower in Myd88^∆Hep^ compared with WT mice. Hence, we cannot rule out that the Myd88^∆Hep^ phenotype may also be due to the indirect effect of *Myd88* deletion on gut microbiota and its metabolites.

In summary, in this study we identify a novel role for hepatic MyD88 in host metabolism. We reveal an unexpected link between this key player of the immune system and liver lipid metabolism independent of its role on inflammation. We demonstrated that in the absence of hepatic *Myd88*, mice were prone to liver fat accumulation and were sensitive to inflammation due to a dysregulation of cholesterol-derivative bioactive lipid synthesis (Fig. 6). Since metabolic syndrome is usually associated with increased liver fat and low-grade inflammation, investigating the exact regulation of MyD88 in the liver might lead to finding some relevant target to tackle metabolic diseases.

## GRANTS

This work is supported by the Funds Baillet Latour (Grant for Medical Research 2015) and European Research Countil Starting Grant 2013 (Starting Grant 336452-ENIGMO). P. D. Cani is a senior research associate at FRS-FNRS (Fonds de la Recherche Scientifique), Belgium. A. Everard is a research associate at FRS-FNRS, Belgium. P. D. Cani is the recipient of grants from FRS-FNRS: under a Projet de Recherche (convention: T.0138.14), FRFS-Walloon Excellence in Life Sciences and Biotechnology (WELBIO) (under Grant WELBIO-CR-2017-C02), and The Excellence of Science (EOS) (EOS 30770923).

## DISCLOSURES

No conflicts of interest, financial or otherwise, are declared by the authors.

## AUTHOR CONTRIBUTIONS

C.L., M.V.H., and P.D.C. conceived and designed research; C.L., M.V.H., A.E., and P.D.C. performed experiments; C.L., M.V.H., A.E., and P.D.C. analyzed data; C.L., M.V.H., A.E., and P.D.C. interpreted results of experiments; C.L. and P.D.C. prepared figures; C.L. and P.D.C. drafted manuscript; C.L., M.V.H., N.M.D., A.E., and P.D.C. edited and revised manuscript; C.L., M.V.H., N.M.D., A.E., and P.D.C. approved final version of manuscript.
